# A Spontaneous *rapZ* Mutant Impairs Infectivity of Lytic Bacteriophage vB_EcoM_JS09 against Enterotoxigenic Escherichia coli

**DOI:** 10.1128/mSphere.01286-20

**Published:** 2021-03-03

**Authors:** Yan Zhou, Hongduo Bao, Hui Zhang, Maoda Pang, Shujiao Zhu, Ran Wang

**Affiliations:** a Institute of Food Safety and Nutrition, Jiangsu Key Laboratory for Food Quality and Safety, State Key Laboratory Cultivation Base, Ministry of Science and Technology, Jiangsu Academy of Agricultural Sciences, Nanjing, China; University of Wyoming

**Keywords:** *rapZ*, lytic bacteriophage, phage resistance, infectivity, enterotoxigenic *E. coli*, LPS, *glmS*

## Abstract

Our understanding of the mechanisms underlying phage-bacterium interactions remains limited. In Escherichia coli, RapZ regulates glucosamine-6-phosphate (GlcN6P) metabolism, the formation of which initiates synthesis of the bacterial cell envelope, including lipopolysaccharides (LPS). However, the role of RapZ, if any, on phage infectivity remains to be investigated. Here, we isolated strains of enterotoxigenic E. coli (ETEC) resistant to its specific lytic bacteriophage vB_EcoM_JS09 (JS09) in a phage aerosol spray experiment. Whole-genome analysis of phage-resistant bacteria revealed the *rapZ* gene acquired a premature stop mutation at amino acid 227. Here, we report that the mutation in the *rapZ* gene confers resistance by inhibiting 93.5% phage adsorption. Furthermore, this mutation changes the morphology of phage plaques, reduces efficiency of plating and phage propagation efficiency, and impairs the infectivity of phage JS09 against ETEC. Using scanning electron microscopy assays, we attribute the inability of the phage to adsorb to the loss of receptors in strains with defective RapZ. Analysis of the LPS profile shows that strains with defective RapZ inhibit phage infection by changing the LPS profile in E. coli. Preincubation of phage JS09 with LPS extracted from a wild-type (WT) strain blocked infection, suggesting LPS is the host receptor for phage JS09 adsorption. Our data uncover the mechanism by which ETEC resists infection of phage JS09 by mutating the *rapZ* gene and then increasing the expression of *glmS* and changing the phage receptor-LPS profile. These findings provide insight into the function of the *rapZ* gene for efficient infection of phage JS09.

**IMPORTANCE** The development of phage-resistant bacteria is a challenging problem for phage therapy. However, our knowledge of phage resistance mechanisms is still limited. RapZ is an RNase adaptor protein encoded by the *rapZ* gene and plays an important function in Gram-positive and Gram-negative bacteria. Here, we report the whole-genome analysis of a phage-resistant enterotoxigenic Escherichia coli (ETEC) strain, which revealed that the *rapZ* gene acquired a premature stop mutation (E227Stop). We show that the premature stop mutation of *rapZ* impairs the infectivity of phage JS09 in ETEC. Furthermore, our findings indicate that ETEC becomes resistant against the adsorption and infection of phage JS09 by mutating the *rapZ* gene, increasing the expression of *glmS*, and changing the phage receptor-LPS profile. It is also first reported here that RapZ is essential for efficient infection of phage JS09.

## INTRODUCTION

Bacteriophages (phages) are bacterium-specific viruses that are able to infect and kill target host bacteria (1–3). Although antibiotics have been widely used to prevent and treat infectious diseases of farm animals for several decades, the overuse and misuse of antibiotics have resulted in bacteria developing resistance to antibiotics (4, 5). As many successful cases for controlling bacterial infections with phages have been reported, phage therapy is considered a promising and potential alternative to antibiotics (4, 6, 7). However, it still has challenging problems to overcome, such as the risk of the development of phage-resistant bacteria (8).

Bacteria can readily evolve phage resistance through diverse mechanisms, including spontaneous mutations (1, 8). It is reported that spontaneous mutations are the main mechanisms driving both phage resistance and phage-bacterium coevolution (8). A crucial first step to a successful phage infection is binding of the virion to receptors on the host cell envelope (9–13). These include lipopolysaccharide (LPS), capsular polysaccharide (CPS), exopolysaccharide (EPS), outer membrane proteins (OMPs), cell wall teichoic acids, and other bacterial cell appendages, such as flagella and pili (8–10, 14, 15). As the main surface component of Gram-negative bacteria, LPS is also the main receptor for phage infection (16, 17). One of the most studied long-tailed myophages, T4, first infects Escherichia coli by gp37 of the long tail fiber reversibly binding OmpC or LPS, and then gp12 of the short tail fiber binds irreversibly to the outer core region of LPS (16, 18). Spontaneous mutations may result in phage resistance by modifying the structure of phage receptors or masking phage receptors (8, 19, 20). Phage-bacterium coevolutionary interactions have been studied for many years (21), yet phage resistance mechanisms still require further elucidation.

Bacteria can avoid or impair phage infection through certain metabolic pathways (1). In E. coli, one metabolic route is the biogenesis of glucosamine-6-phosphate (GlcN6P), an early and essential precursor in the synthesis of the bacterial cell envelope constituents, such as peptidoglycan, LPS, and colanic acid (22–25). In both Gram-positive and Gram-negative bacteria, RapZ plays a central role in positive and negative feedback that regulates GlcN6P metabolism (26). RapZ (formerly YhbJ) is a 32.49-kDa RNase adaptor protein encoded by the *rapZ* gene (27). It has a C-terminal RNA-binding domain, which cooperates with small RNAs (sRNAs) GlmY and GlmZ to regulate enzyme GlmS (GlcN6P synthase) mRNA (28, 29). RapZ strongly affects the expression of *glmS*, and GlmS synthesizes GlcN6P, which is required for the biosynthesis of LPS (22, 25, 28). The C-terminal domain of RapZ senses and binds GlcN6P with high specificity (25, 30). Recent studies showed that in phage-resistant bacteria of E. coli K-12 and Staphylococcus aureus SA003, mutations in *rapZ* are found by genomic analysis, suggesting that RapZ is involved in phage resistance (31, 32), although the full mechanism remains to be elucidated.

Lytic phage vB_EcoM_JS09 (JS09) was isolated from sewage samples in China, and the genomic and biological characteristics of this phage were reported by our group (33). It has a broad host range and infects antibiotic-resistant enterotoxigenic E. coli (ETEC) and avian pathogenic E. coli (APEC). It has been fully sequenced (GenBank accession number KF582788), revealing a genome of 169.148 kb with 273 open reading frames (ORFs) as the basis of coding sequences (CDS). JS09 is a T4-like phage that infects its host ETEC strain EK99-F41, causing rapid and persistent bacterial cell lysis for more than 24 h (33). The putative short tail fiber protein gp12 of JS09 contains a conserved short tail fiber receptor-binding domain, which has a potential LPS-binding site. In our phage aerosol spray experiment, we isolated several spontaneous phage-resistant bacterial mutants of EK99-F41. In this work, we use whole-genome sequencing of EK99-F41 and its phage-resistant mutants to identify and study genes essential for phage JS09 infectivity.

On the other hand, the interactions between phages and bacterial genes still need to be clarified (34). In the present study, we identified that a spontaneous *rapZ* mutant impairs the infectivity of lytic phage JS09 against ETEC. We also demonstrated that the premature stop mutation of *rapZ* inhibited JS09 infection by increasing the expression levels of *glmS* and changing the LPS profile in ETEC. This work will be helpful for understanding the phage resistance mechanisms and preventing the risk of developing phage resistance in phage therapy.

## RESULTS

### Whole-genome analysis of phage-resistant strain WRP.

**FIG 1:**
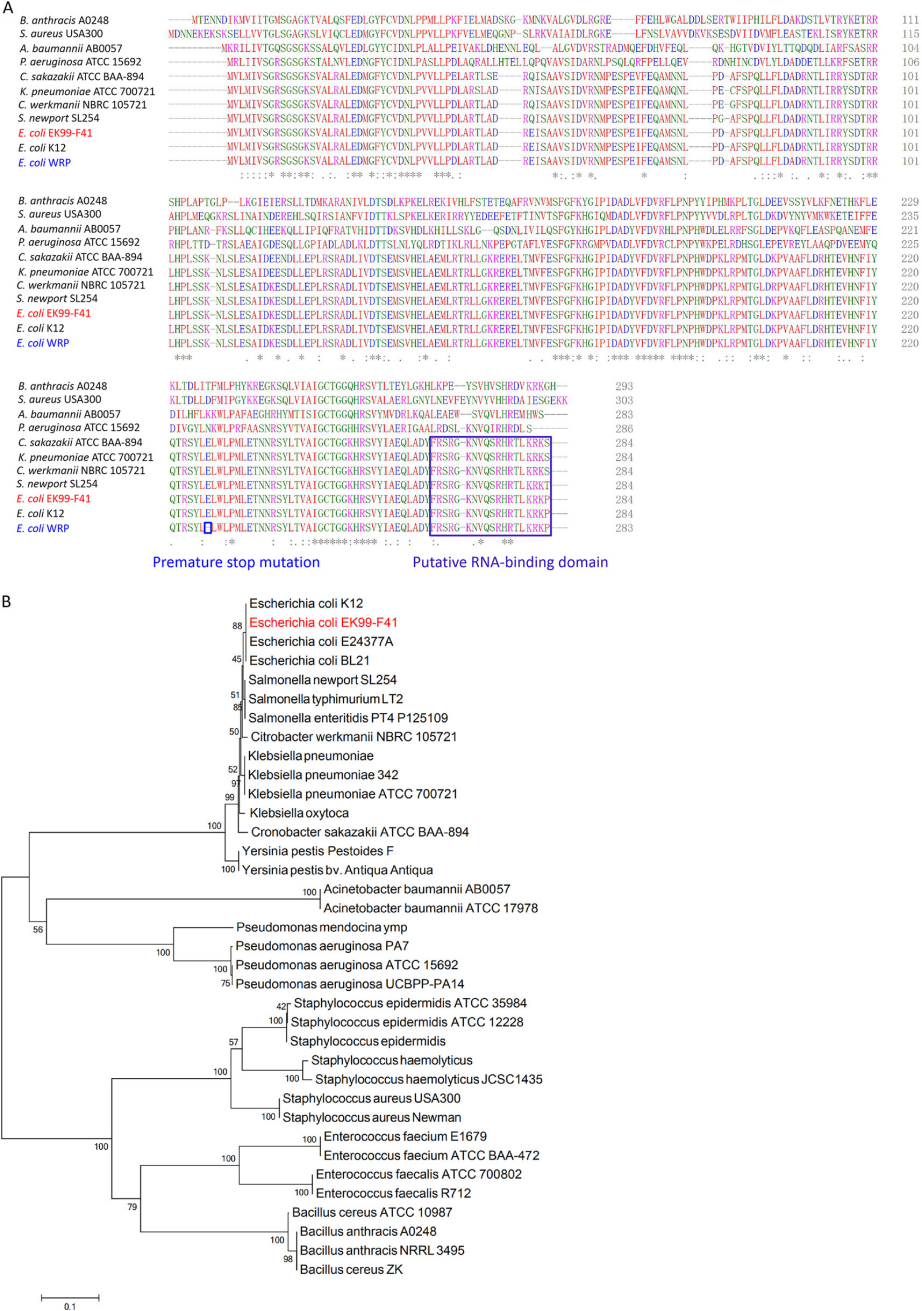
RapZ is widely distributed in bacteria. (A) Multiple-amino-acid-sequence alignment (by Clustal Omega) of 11 different bacterial RapZ proteins. Identical amino acid residues (aa) are marked with an asterisk (*), strongly similar aa are marked with two dots (:), and weakly similar aa are marked with dot (.). The premature stop mutation site in WRP and putative RNA-binding domain of RapZ are indicated by a blue square and a purple square, respectively. (B) Phylogenetic analysis of 36 RapZ proteins from different bacteria. The phylogenetic tree was constructed using the neighbor-joining method. An additional 35 RapZ sequences were collected from UniProtKB and used for phylogenetic tree construction using MEGA 6.0 with neighbor-joining method with a bootstrap replicate number of 1,000.

To determine the genome changes associated with phage resistance, we performed whole-genome sequencing of select spontaneous phage-resistant strain WRP and corresponding wild-type (WT) parental strain EK99-F41 (Table 1). The whole genomes of WRP and EK99-F41 were sequenced on an Illumina HiSeq 2500 platform. Mutations detected in the sequenced strains were mapped against the WT EK99-F41 reference genome. Compared to EK99-F41, a total of four nucleotide mutation sites were identified in WRP (Table 2). Three sites were nonsynonymous substitutions, which are located in ORFs encoding F-type conjugal transfer pilus assembly protein TraB, DNA polymerase II, and glucose-specific phosphotransferase enzyme IIA component. One-point mutation (GAG→TAG) in *rapZ*, which encodes the RNase adaptor protein RapZ, was a premature stop mutation (Fig. 1A). The premature stop mutation within RapZ accounted for a truncation of the C-terminal 58 amino acids (RapZ amino acids 227 to 284). The region from amino acids 266 to 284 is a putative RNA-binding domain (23) (Fig. 1A). A phylogenetic tree of RapZ proteins of various bacteria revealed the close relationship of EK99-F41 to E. coli K-12 (100% identity) and related strains (Fig. 1B). RapZ indirectly participates in the assembly of cell wall components, including peptidoglycan and LPS in E. coli (22, 24). It is also involved in cell envelope integrity (25). Therefore, we speculated that the mutation in *rapZ* contributed to phage resistance.

**TABLE 1:** Bacterial strains, phage, and plasmids used in this study

Strain, phage, or plasmid	Description	Reference or source
Strains		
E. coli WT EK99-F41	ETEC, 81.8% genome sequence identity with E. coli strain 6409	33
E. coli DH5α	ϕ80d *lacZ*ΔM15 *recA1 endA1 gyrA96 thi-1 hsdR17* (r_K_^−^ m_K_^+^) *supE44 relA1 deoR* Δ(*lacZYA-argF*) *U169*	Laboratory stock
E. coli WRP	A phage-resistant mutant of E. coli WT EK99-F41 from phage aerosol spray expt	This work
E. coli *rapZ*_E227Stop_	Same as E. coli WT EK99-F41, but *rapZ* with amino acid E227-stop codon mutation (i.e., nucleotide G679T mutation)	This work
C-*rapZ*_E227Stop_	Complemented E. coli *rapZ*_E227Stop_, transformed with a pACYC177 vector inserted with cloned *rapZ* gene from EK99-F41	This work
Phage		
E. coli phage vB_EcoM_JS09	ETEC lytic phage, GenBank accession no. KF582788	33
Plasmids		
pACYC177	Low-copy-number cloning vector, Kan^r^	60
pACYC177::RapZ	Cloning vector with a PCR fragment covering *rpoN* operon promoter, RBP region, and the *rapZ* gene in the BamHI/PstI site; Kan^r^	This work

**TABLE 2:** Genes with mutations in WRP compared to WT EK99-F41

Gene	Position in the genome (nt)^*a*^	Gene product	Proposed function	Protein length (aa)^*b*^	Mutation position in the gene (nt)	Mutation codon^*c*^	Mutation amino acid
EK99-F41004252 (−)	12155–13519	F-type conjugal transfer pilus assembly protein, TraB	Environmental information processing, membrane transport, secretion system	454	79	GGG→aGG	G27R
EK99-F41002631 (−)	74602–75456	RNase adapter protein, RapZ	GlmZ (sRNA)-inactivating NTPase	284	679	GAG→tAG	Nonsense (E227-stop codon)
EK99-F41001465 (−)	113847–116198	DNA polymerase II, PolB	DNA repair and recombination	783	1652	GTC→GcC	V551A
EK99-F41000289 (+)	329347–329856	Glucose-specific phosphotransferase enzyme IIA component	Carbohydrate metabolism	169	3	ATG→ATt	M1I

a nt, nucleotide.

b aa, amino acid.

c Mutated nucleotide residues are lowercase.

### *rapZ* is requires for phage adsorption.

**FIG 2:**
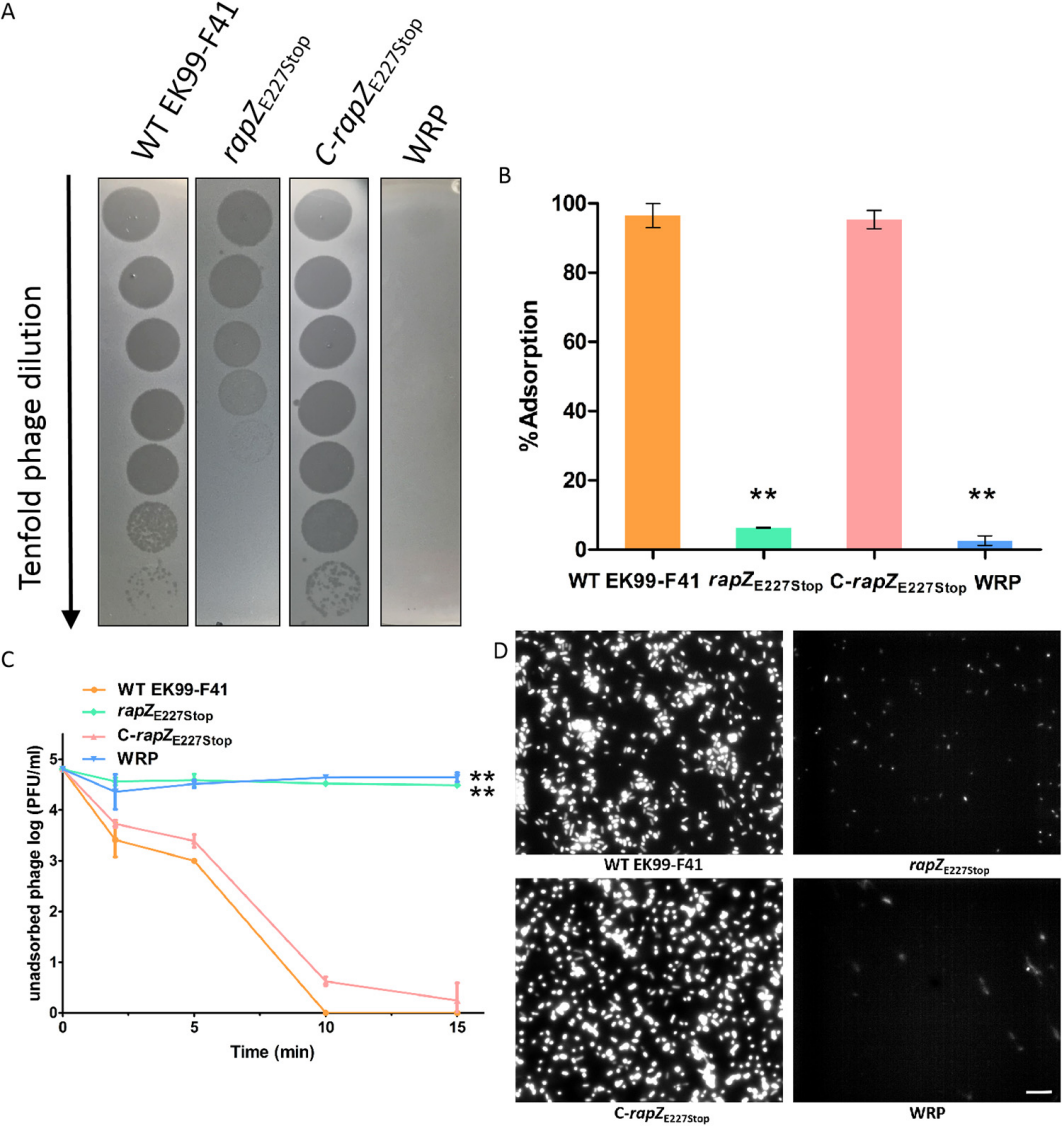
*rapZ* is required for phage adsorption. (A) Tenfold dilution of lysates of phage JS09 applied to bacterial lawns of WT EK99-F41, *rapZ*_E227Stop_, C-*rapZ*_E227Stop_, and WRP strains. Clear zones indicate cell death. Shown are representative images from at least three independent experiments. (B) Phage JS09 adsorption to WT EK99-F41, *rapZ*_E227Stop_, C-*rapZ*_E227Stop_, and phage-resistant mutant WRP strains, shown as percentage of adsorbed phage. The strains used for adsorptions are presented along the *x* axis (complete strain names and descriptions can be found in Table 1). Error bars show standard deviations. Significance was determined by Student’s *t* test for comparison between the mutant group and the WT group. **, *P* < 0.01. (C) Adsorption kinetics of phage JS09 to WT EK99-F41, *rapZ*_E227Stop_, C-*rapZ*_E227Stop_, and WRP strains, shown as percentage of residual phage. Error bars show standard deviations. Significance was determined by Student’s *t* test for comparison between the mutant group and the WT group. **, *P* < 0.01. (D) Visualization of phage adsorption to the surfaces of the four bacterial strains by DAPI-labeled JS09 phages under a Zeiss Scope A1 epifluorescence microscope. Bar, 2 μm.

To determine the roles of RapZ in phage resistance, we constructed the same premature stop mutation, generating the *rapZ*_E227Stop_ mutant strain. To avoid the mutant phenotype caused by a polar effect or a spontaneous mutation elsewhere in the genome, we also constructed C-*rapZ*_E227Stop_, i.e., *rapZ*_E227Stop_ mutant that was complemented in *trans* by the corresponding wild-type gene (Table 1). As shown in Fig. 2, phage JS09 susceptibility of WT and mutant strains was assayed. JS09 formed plaques very faintly on the WRP mutant and opaquely without clarity on the *rapZ*_E227Stop_ mutant compared to those on the WT EK99-F41 strain (Fig. 2A). The results suggest that under identical culture conditions, the phage resistance of WRP is higher than that of the *rapZ*_E227Stop_ mutant. The *rapZ*_E227Stop_ mutant did not completely alter the ability of the phage to lyse the bacteria; however, its susceptibility to JS09 phage was reduced. In the transcomplementation assay, introduction of *rapZ* (pACYC177::RapZ) into *rapZ*_E227Stop_ restored its sensitivity to phage JS09 (Fig. 2A).

A phage adsorption assay revealed that more than 95% of the phages were rapidly adsorbed onto the host cells of EK99-F41 and C-*rapZ*_E227Stop_ strains within 15 min (Fig. 2B). In contrast, the *rapZ*_E227Stop_ and WRP strains had inhibited phage adsorption to receptors on the bacterial surface (Fig. 2B and C). *rapZ*_E227Stop_ and WRP strains had JS09 binding reduced by 93.5% and 97.3%, respectively (Fig. 2B), suggesting the phage receptors are affected by the *rapZ*_E227Stop_ mutation. These results indicated that bacterial *rapZ* is important and required for successful adsorption and infection by phage JS09.

To further examine the phage adsorption, 4′,6-diamidino-2-phenylindole (DAPI)-labeled JS09 phages were incubated with the four bacterial strains. Fluorescently labeled phage JS09 attached to the sensitive cells of WT EK99-F41 and the C-*rapZ*_E227Stop_ strain, whereas attachment of a few phages was observed in *rapZ*_E227Stop_ and WRP strains (Fig. 2D).

### *rapZ*_E227Stop_ mutant impairs infectivity of phage JS09.

**FIG 3:**
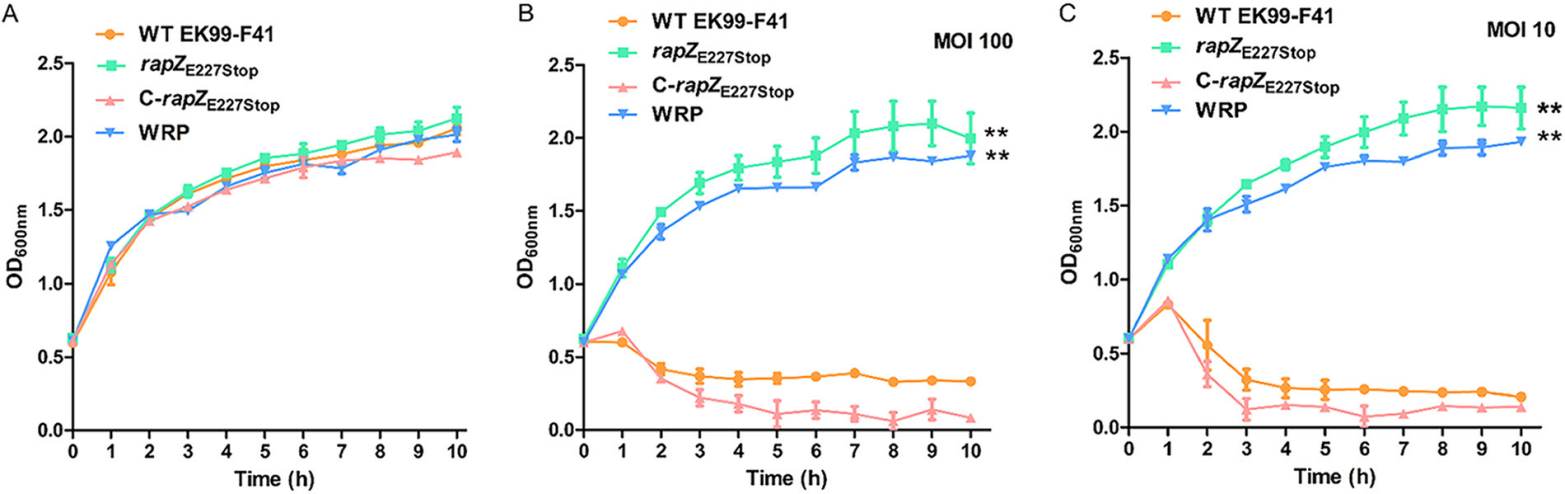
*rapZ*_E227Stop_ mutant impairs infectivity of phage JS09. (A) Bacterial growth kinetics. Error bars represent standard deviations from triplicate samples; some are hidden by the symbols. (B) Infectivity assay of WT EK99-F41, *rapZ*_E227Stop_, C-*rapZ*_E227Stop_, and phage-resistant mutant WRP strains infected with JS09 phages at an MOI of 100. Error bars represent standard deviation from triplicate samples; some are hidden by the symbols. Significance was determined by Student’s *t* test for comparison between the mutant group and the WT group. **, *P* < 0.01. (C) Infectivity assay of WT EK99-F41, *rapZ*_E227Stop_, C-*rapZ*_E227Stop_, and phage-resistant mutant WRP strains infected with JS09 phages at an MOI of 10. Cell lysis was followed by OD_600_ measurements at the indicated time points. Error bars represent standard deviations from triplicate samples; some are hidden by the symbols. Significance was determined by Student’s *t* test for comparison between the mutant group and the WT group. **, *P* < 0.01.

To determine whether the premature stop mutation of *rapZ* affected the infectivity of phage JS09, we incubated bacterial strains with JS09 using the phage infection assay. As shown in Fig. 3A, all four bacterial strains had the same growth curves within 10 h. By coincubating phage JS09 with these strains, the phage significantly inhibited the growth of WT EK99-F41 and the C-*rapZ*_E227Stop_ strain. The optical density at 600 nm (OD_600_) of WT EK99-F41 decreased from 0.6 to 0.23 and 0.28 after a 3-h infection with phage at multiplicities of infection (MOIs) of 100 and 10, respectively. In comparison with WT EK99-F41, we did not detect the lytic activity of JS09 against the *rapZ*_E227Stop_ and WRP strains in liquid culture (Fig. 3B and C), suggesting the infectivity of phage JS09 is impaired by the premature stop mutation of *rapZ*. Taken together, the *rapZ*_E227Stop_ mutant exhibited resistance to phage JS09. Since the premature stop mutation of *rapZ* in EK99-F41 impaired phage infectivity to the same extent as that observed for WRP, we did not investigate the effects of the other three mutant genes identified as described above on phage resistance in this study.

### *rapZ*_E227Stop_ reduces phage propagation.

**FIG 4:**
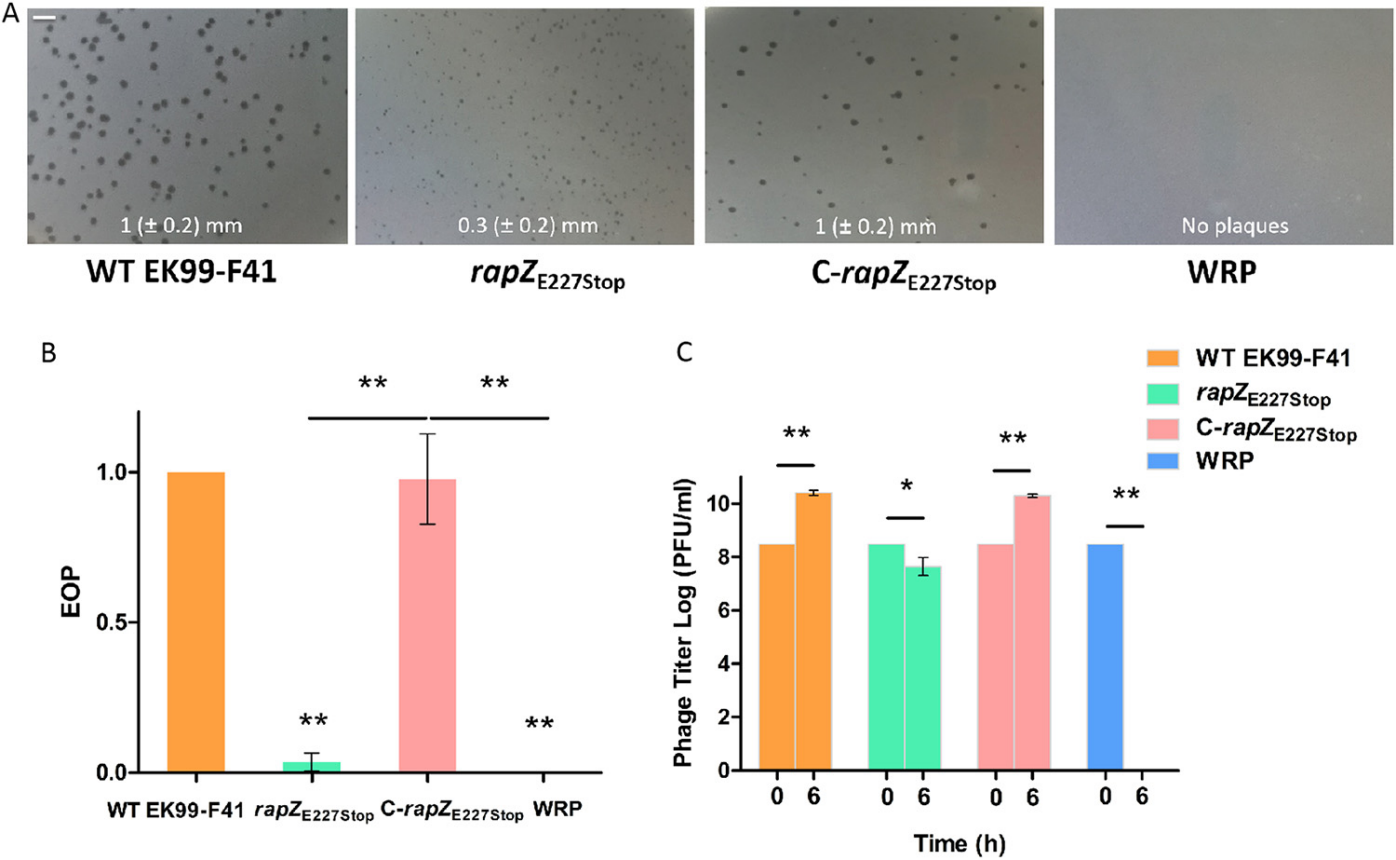
Phage JS09 propagation on the four bacterial strains. JS09 phages (∼10^10^ PFU/ml) were utilized to infect WT EK99-F41, *rapZ*_E227Stop_, C-*rapZ*_E227Stop_, and WRP strains. (A) Shown are representative plaque images and the average plaque diameters, which were calculated from at least 30 plaques from three different independent experiments. Only unambiguous plaques from each experiment were considered for the analysis. Bar, 4 mm. (B) The number of plaques was monitored 6 h postinfection. Shown is the number of plaques obtained on each strain divided by the number of plaques obtained on WT EK99-F41 cells, i.e., EOP (efficiency of plating) on each strain. Shown are average values and standard deviations (SDs) from at least three independent experiments. Significance was determined by Student’s *t* test. **, *P* < 0.01. (C) Phage propagation was detected by the double agar overlay plaque assay. The number of plaques was monitored 6 h postinfection. Shown is the number of plaques obtained on each strain. Shown are average values and SDs from at least three independent experiments. Significance was determined by Student’s *t* test. *, *P* < 0.05; **, *P* < 0.01.

To further characterize phage propagation in mutant strains, phage JS09 was used to infect WT EK99-F41, *rapZ*_E227Stop_, C-*rapZ*_E227Stop_, and WRP strains in liquid medium. For the controls, phages were able to form clear plaques of the same size on WT EK99-F41 and C-*rapZ*_E227Stop_ cells (Fig. 4A). In comparison, plaques formed on the *rapZ*_E227Stop_ mutant were significantly smaller and opaque (Fig. 4A). For the *rapZ*_E227Stop_ mutant, the efficiency of plating (EOP) was greatly reduced (Fig. 4B). The number of plaques formed on a *rapZ*_E227Stop_ lawn decreased 96.5% in comparison to those formed on a WT lawn (Fig. 4B). Although JS09 phage was able to form plaques on the *rapZ*_E227Stop_ mutant, the phage propagation efficiency declined and the infection led to negative propagation (Fig. 4C). No plaques formed on the WRP lawn, demonstrating that WRP is stable and resistant to phage JS09 (Fig. 4B and C). These results support the view that *rapZ*_E227Stop_ impairs the infectivity of phage JS09.

### *rapZ*_E227Stop_ alters the bacterial morphology.

**FIG 5:**
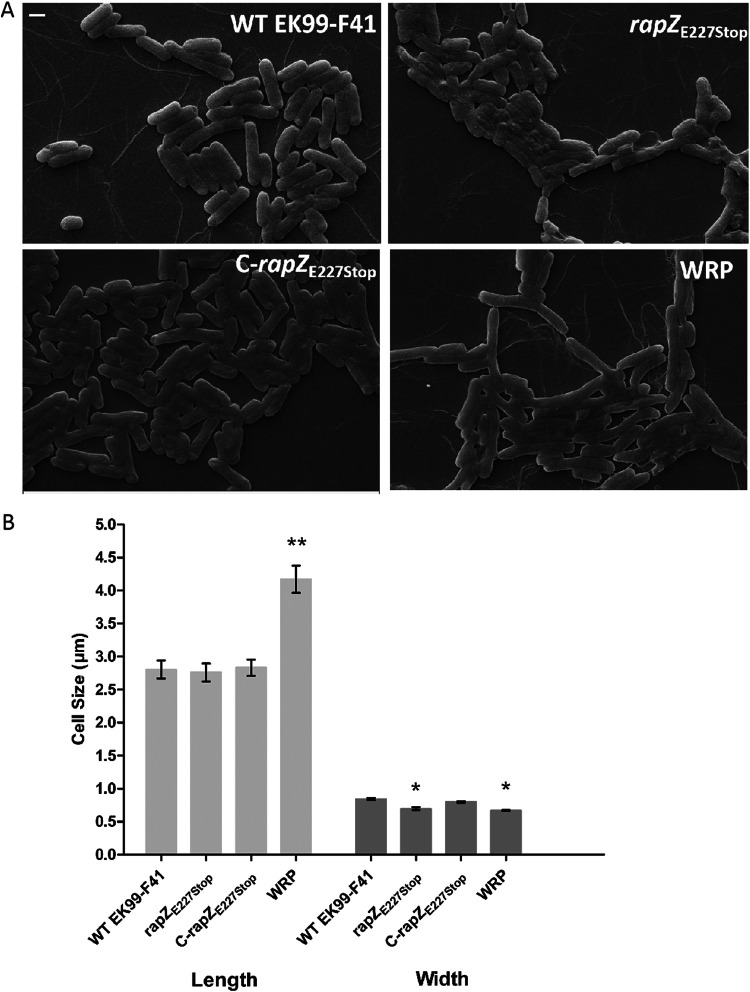
Influences of *rapZ*_E227Stop_ on cell morphology. (A) SEM images of WT EK99-F41, *rapZ*_E227Stop_, C-*rapZ*_E227Stop_, and WRP strains. Bar, 1 μm. (B) Measurements of cell dimensions (40 cells per strain). The experiments were independently replicated three times. Error bars show standard deviations. Significance was determined by Student's *t* test for comparison between the mutant group and the WT group. *, *P* < 0.05; **, *P* < 0.01.

We speculated that the cell surface structure of bacterial mutants might have changed and inhibited phage adsorption. To test this hypothesis, we observed the bacterial cell morphology by scanning electron microscopy (SEM). SEM image analysis revealed that the cell surface of the *rapZ*_E227Stop_ mutant was rough and appeared damaged, and the cell shape of WRP became irregular (Fig. 5A). The average length of the *rapZ*_E227Stop_ cells was the same as that of the EK99-F41 cells, while the average widths of the *rapZ*_E227Stop_ and WRP cells were 16.7% and 20.2% thinner than that of the EK99-F41 cells, respectively (Fig. 5B). The bacterial morphology was restored in the C-*rapZ*_E227Stop_ strain. However, the average length of the WRP cells was 48.9% longer than that of the EK99-F41 cells. This phenomenon is probably related to the other three gene mutations in WRP. Nonetheless, these data indicated that RapZ is required to maintain bacterial cell envelope integrity.

### *rapZ*_E227Stop_ inhibits phage infection by changing the LPS profile in E. coli.

**FIG 6:**
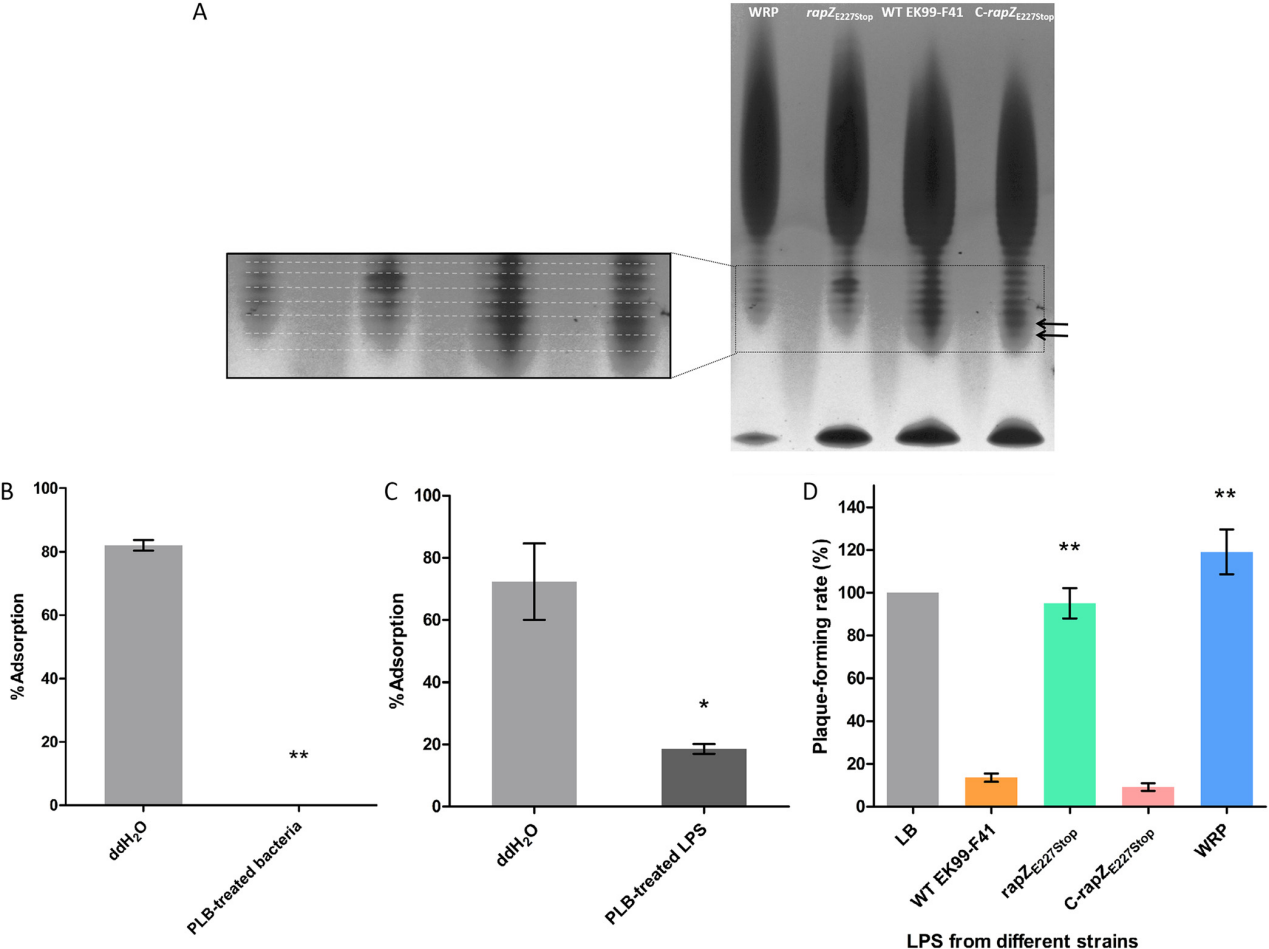
Effects of LPS extracted from each strain on JS09 infection of WT EK99-F41. (A) LPS profiles of the four bacterial strains by silver-stained SDS-PAGE analysis. The experiments were replicated in triplicates, and a representative image is shown. Effect of PMB on adsorption of phage JS09 to WT EK99-F41 cells (B) and LPS extracted from WT EK99-F41 (C), shown as percentage of adsorbed phage. Treatment was with 25 μg/ml PMB for 1 h at 37°C. For the untreated group, double-distilled water was used as a control. (D) Competitive binding assay is shown by plaque-forming rates of JS09 preincubated with LPS extracted from different strains. PFU of the group without LPS (gray bar) was set as 100%. The experiments were independently replicated three times. Error bars show standard deviations. Significance was determined by Student's *t* test for comparison between the mutant group and the WT group. *, *P* < 0.05; **, *P* < 0.01.

To determine if the premature stop mutation of *rapZ* influences the synthesis of LPS, we compared the LPS profiles of WT EK99-F41, *rapZ*_E227Stop_, C-*rapZ*_E227Stop_, and WRP strains. For consistency, we extracted LPS from the same amounts of bacterial cultures. In agreement with the phenotypes (Fig. 2A), the WT EK99-F41 and C-*rapZ*_E227Stop_ strains produced similar LPS patterns (Fig. 6A). In contrast, the amount of LPS in the WRP mutant was decreased, and both WRP and *rapZ*_E227Stop_ strains appeared to show deficiency in LPS structures, likely in O-antigen (35).

To determine if LPS of WT EK99-F41 is the receptor of phage JS09, we used polymyxin B (PMB) to pretreat bacterial cells and extracted LPS. Polymyxin B (PMB) is a cationic antibiotic that specifically binds and neutralizes the LPS of Gram-negative bacteria (36–38). By modifying LPS structure, PMB blocks the adsorption of phage to the LPS receptor (36, 38). Similarly, PMB can neutralize the phage binding and inactivation ability of isolated LPS from E. coli strain Nissle 1917 (EcN) (37). We tested phage adsorption to WT EK99-F41 LPS by pretreating either bacterial cells or extracted LPS with and without 25 μg/ml PMB for 1 h at 37°C and then coincubating with JS09 phage. We observed complete loss of adsorption of JS09 to WT EK99-F41 bacterial cells after treatment with 25 μg/ml PMB (Fig. 6B). We also found that the phage adsorption efficiency of extracted LPS from WT EK99-F41 was greatly reduced by treatment with 25 μg/ml PMB (Fig. 6C).

In addition to the differences of LPS profiles, we also tested the effect of the extracted LPS on the infection by JS09. As shown in Fig. 6D, preincubation of JS09 with the LPS extracted from WT EK99-F41 and C-*rapZ*_E227Stop_ strains greatly reduced the efficiency of plating (EOP), whereas the LPS extracted from *rapZ*_E227Stop_ and WRP strains showed no such effect. Apparently, blocking JS09 with the extracted LPS from WT EK99-F41 and C-*rapZ*_E227Stop_ strains significantly inhibited the efficiency of infection. Due to the influences of the premature stop mutation of *rapZ* on LPS, the extracted LPS from *rapZ*_E227Stop_ and WRP strains did not block the infection of JS09. Meanwhile, these data also revealed that LPS is the host receptor for phage JS09 adsorption.

### *rapZ*_E227Stop_ increases the expression of *glmS* in E. coli.

**FIG 7:**
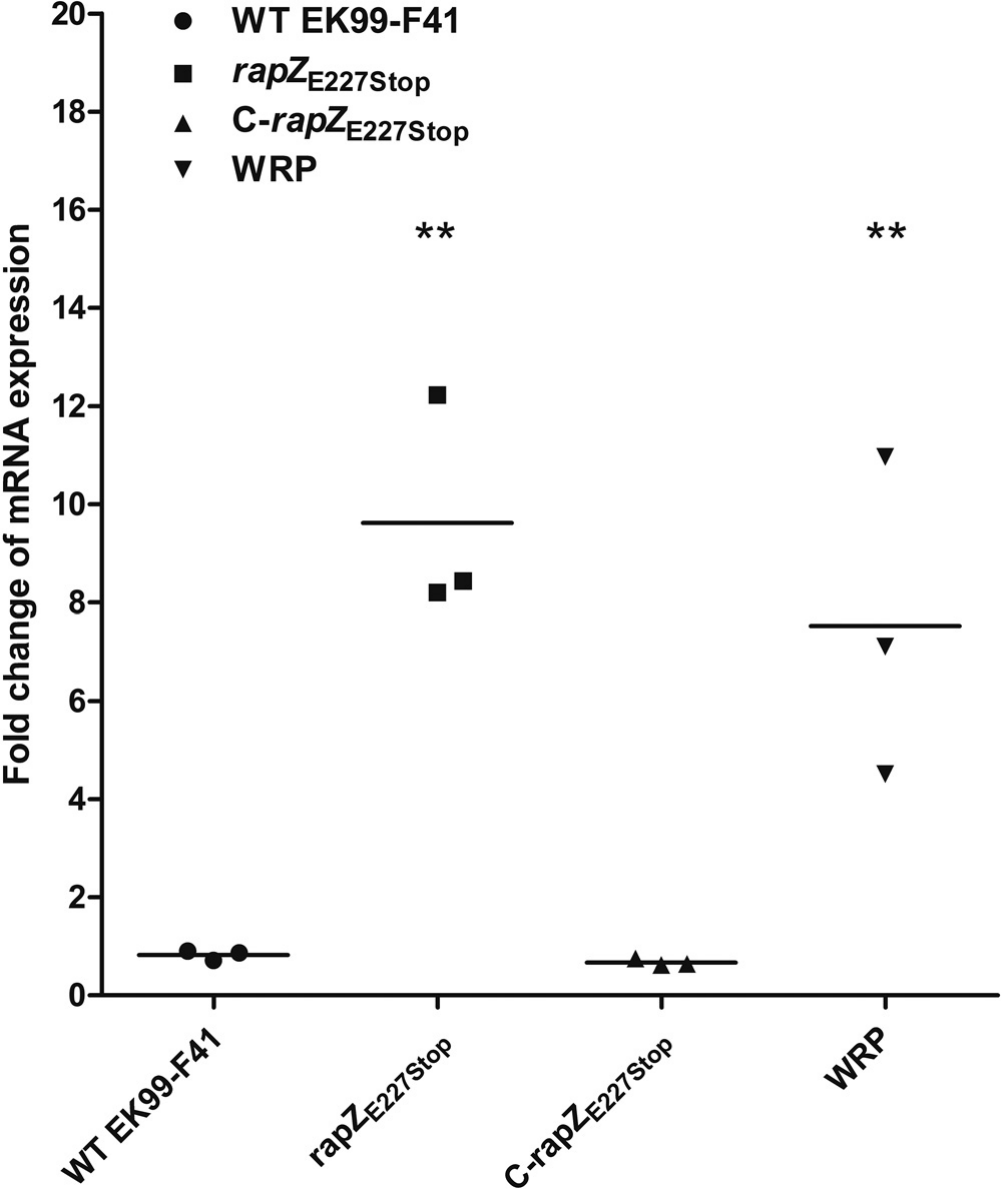
Analysis of *rapZ*_E227Stop_ effects on expression of *glmS*. *rapZ*_E227Stop_ affects expression of *glmS* in E. coli. The transcript levels of *glmS* were analyzed by qRT-PCR. The expression of the *glmS* gene was significantly increased in *rapZ*_E227Stop_ and WRP strains. Error bars show standard deviations. Significance was determined by Student’s *t* test for comparison between the mutant group and the WT group. **, *P* < 0.01.

The mutant defective in *rapZ* was previously demonstrated to lead to the derepression of *glmS* transcript and overproduction of GlmS (22). Moreover, it has been verified that RapZ binds specifically to GlcN6P (25). We hypothesized that bacteria became resistant to phage infection by mutating the *rapZ* gene, resulting in the emergence of the *rapZ*_E227Stop_ gene. Due to the defective C-terminal domain, RapZ_E227Stop_ was not able to bind GlmY, GlmZ, or GlcN6P. Furthermore, RapZ_E227Stop_ lost the ability to sense GlcN6P scarcity, which would lead to insufficient synthesis of LPS. LPS acted as the adsorption receptor for phage infection; thus, phages could not be completely and effectively adsorbed to and infect bacteria. However, GlcN6P is essential to bacterial growth, which would cause the accumulated expression of *glmS*. Hence, we sought to evaluate whether the changes of *glmS* expression influence the LPS profile in mutant strains. Compared to that in WT EK99-F41 and C-*rapZ*_E227Stop_ strains, the expression of the *glmS* gene in *rapZ*_E227Stop_ and WRP strains was significantly increased (Fig. 7). These data demonstrated that ETEC resists infection by phage JS09 through mutation of the *rapZ* gene and then increases the expression of *glmS* and changes the phage receptor-LPS profile.

## DISCUSSION

Due to the emergence and development of antibiotic-resistant bacteria, it has become increasingly difficult and expensive to treat bacterial infections (39–41). Thus, phage therapy is attracting attention for the treatment of human and animal infectious diseases. As such, the appearance of phage-resistant bacteria is a pending problem in phage therapy (8). However, there are still many unknowns about the molecular mechanism behind the phage resistance phenomenon. To fully understand the phage resistance mechanism, in this study, we identified that a premature stop mutation at amino acid 227 in the protein encoded by *rapZ* reduced phage susceptibility by inhibiting phage JS09 adsorption to ETEC, thereby decreasing phage propagation. Although the *rapZ*_E227Stop_ strain was still susceptible to phage JS09 (10^7^ to 10^10^ PFU per ml), infectivity of phage JS09 was significantly decreased. Furthermore, our results indicated that *rapZ* mutant strains are resistant to infection by phage JS09 by increasing the expression of *glmS* and changing the phage receptor-LPS profile, supporting the prediction that ETEC impairs the infectivity of lytic phage JS09 through mutation of *rapZ*.

A few researches have reported the connection of RapZ and phage resistance (31, 32). RapZ is widespread in various bacteria and belongs to a family of RNA-binding proteins. In E. coli K-12, the RNA-binding domain of RapZ is predicted in the region of amino acids 266 to 284 (23). It is demonstrated that a RapZ variant carrying the mutation K270A-K281A-R282A-K283A in the RNA-binding domain failed to bind GlmY and GlmZ (23). The RNA-binding domain of RapZ is conserved not only in E. coli, but also in Salmonella enterica serovar Newport, S. enterica serovar Typhimurium, Citrobacter werkmanii, Cronobacter sakazakii, Klebsiella pneumoniae, and Yersinia pseudotuberculosis (23). In S. aureus, RapZ also modulates the expression of GlmS, which is the key enzyme that feeds glucose into cell wall synthesis (31). Azam et al. found that spontaneous point mutations in *rapZ* inhibited phage adsorption in phage-resistant S. aureus (31). The amino acid mutations of RapZ in S. aureus were P48A and H267D (31). Afterwards, Mutalik et al. reported that in E. coli K-12 *rapZ* multicopy expression conferred resistance against N4 phage (32). These further demonstrate that phage infection caused the occurrence of phage-resistant bacteria by mutations in the *rapZ* gene. In agreement, a *rapZ*_E227Stop_ mutant, which was a C-terminally truncated RapZ (amino acids 1 to 226), lacking the RNA-binding domain, exhibited phage resistance through inhibition of phage adsorption. Furthermore, for the first time, our findings confirmed that mutation of *rapZ* not only inhibited 93.5% of phage adsorption but also impaired the infectivity of lytic phage JS09 against ETEC.

Bacteria have evolved a variety of strategies to prevent phage adsorption (42). Adsorption of phages to host receptors is the essential and initial step of infection (13). Hence, bacterial outer membrane proteins and cell envelope components involved in adsorption-blocking mechanisms have been well studied (13, 42, 43). However, little is reported on the connection between bacterial metabolic route and adsorption-blocking mechanisms. RapZ directly regulates the metabolism of GlcN6P, which begins the synthesis of the bacterial cell envelope (25). In this work, we confirmed that *rapZ*_E227Stop_ mutant strains inhibit phage adsorption. The results may suggest that bacteria inhibit phage adsorption by mutating the *rapZ* gene to change the bacterial surface morphology.

RapZ is highly conserved in bacteria, plays a crucial role in the bacterial physiology state, and directly influences the formation of the bacterial cell envelope (25). Therefore, we examined the changes of bacterial morphology by SEM. In phage-resistant S. aureus strains, it was suggested that spontaneous mutations of the *rapZ* gene caused increased capsular polysaccharide production (31). Overproduction of capsular polysaccharide blocks phage receptors on the cell surface and affects biofilm formation (19, 32, 44). However, in this study, we found that *rapZ*_E227Stop_ and WRP strains did not show promoted biofilm formation or a change in bacterial motility (data not shown). Considering the damage of the cell envelope observed by SEM (Fig. 5A), we speculate that *rapZ*_E227Stop_ affected the receptors necessary for adsorption by phage JS09, thus providing the mechanism for bacterial resistance to phage infection.

LPS functions as the essential permeability barrier in Gram-negative bacteria (45). LPS biosynthesis is complicated, and in this process, any defects or any imbalance will cause major cellular defects (45). It is reported that the main host receptor in Gram-negative bacteria is LPS (18). In our published paper, phage JS09 belongs to the *Caudovirales* order (*Myoviridae* phage family) and is considered a T4-like phage (33). Therefore, we speculated that LPS functions as the receptor of phage JS09 in the process of reversible or irreversible adsorption. In the reversible adsorption process of E. coli K-12 phage Bp7 with LPS mutant strains, an obvious periodic phage binding-release cycle can be observed within 15 min (46). However, we did not observe that cycle in phage adsorption assays with *rapZ*_E227Stop_ mutant strains (Fig. 2C). On the basis of the plaque formation results (Fig. 2A), the *rapZ*_E227Stop_ mutant did not completely inhibit phage infection. It is reported that reversible adsorption is not necessary for phage infection (47). Therefore, our results indicate that *rapZ*_E227Stop_ mutants inhibited the reversible adsorption of phage JS09.

Detection of the LPS profile has been demonstrated to be a feasible tool for screening phage-resistant mutants (35). Based on the LPS profiling method, it is effective to screen for E. coli 4s phage-resistant spontaneous mutants with impaired or altered LPS synthesis (35). It remains unknown whether the changes to the LPS profile in bacteria are related to RapZ. As shown in Fig. 7, the expression of *glmS* was increased in mutant strains. We hypothesized that *rapZ*_E227Stop_ caused these changes. Mutations defective in RapZ increase the expression of *glmS* and result in overproduction of GlmS (22, 28). Likewise, depletion of the intracellular GlcN6P induces accumulation of the *glmS* transcript and GlmS synthesis (22). As to the proliferation of *rapZ*_E227Stop_ mutant strains, the lack of intracellular GlcN6P would lead to less synthesis or a variation of LPS. Thus, our investigations suggest a direct correlation between LPS and RapZ.

It remains to be determined whether RapZ cooperates with other bacterial pathways in the phage infection mutants. RapZ interacts with the bacterial quorum sensing (QS) system, QseE/QseF, and stimulates their phosphorylation (25). The QS system plays crucial roles in many bacterial cellular pathways, and it can render bacteria resistant to phage infection via reducing the amount of phage receptors on the cell surface (13, 14). Additionally, the QS system can attenuate phage reproduction by affecting the bacterial physiological state and cell populations (48). Similarly, we also found the phage titer was reduced when phage JS09 was propagated in the *rapZ*_E227Stop_ mutant compared to the original phage titer (Fig. 4B and C). Finally, the *rapZ*_E227Stop_ mutant showed an abnormal cell state (Fig. 5). Remarkably, Khan et al. identified RapZ at the heart of bacterial cell envelope precursor metabolite sensing and signaling (25). This further suggests that phage infection is associated with the QS system and bacterial metabolism. Next, we will focus on a comparison of the metabolic products between WT EK99-F41 and *rapZ*_E227Stop_ strains.

The relationship of bacteria and phages is parasitic, and so the arms race between them leads to their coevolution (1, 49). Although phage therapy is a promising alternative to antibiotics, the function of bacterial proteins involved in phage infection remains limited (14). Consistent with Görke’s group findings on RapZ (24–26, 29), our data also imply that RapZ is involved in the synthesis of the cell envelope. Consequently, defining the phage resistance mechanisms could help to further the understanding of the function of bacterial genes and contribute to the development of phage therapy by improving the design of effective phage formulas.

It should be noted that in this work we concentrate on only *rapZ*. The residual infectivity seen in the *rapZ*_E227Stop_ strain but not WRP shows it is still susceptible to phage JS09; one possible reason is that there might be three additional mutants cooperatively determining the phage infection efficiency. For the other three mutant genes in WRP (Table 2), TraB is an F-type conjugal transfer pilus assembly protein and is involved in pilus extension (50). F-pilus contains specific regions that can be associated with filamentous phage sensitivity (50). Except filamentous phages, the interaction of F-pilus and other types of phages is unknown. DNA polymerase II is a member of the B family of DNA polymerases and is involved in both replicative and reparative processes, particularly in 5′ to 3′ DNA-dependent DNA polymerase activity and in 3′ to 5′ exonuclease proofreading activity (51). Glucose-specific phosphotransferase enzyme IIA component (IIA^Glc^), a component of the phosphotransferase system (PTS) of E. coli, is important in regulating carbohydrate metabolism, and its state is of importance for the regulation of cell growth (52). However, no one had published on the relationship between those two genes and phages. Our further work will investigate the influence of those three genes on phage infectivity.

In summary, our work presented here provides further experimental evidence that the premature stop mutation in *rapZ* results in the emergence of phage resistance. More importantly, RapZ is required for effective phage infection by presenting the phage receptors in E. coli.

## MATERIALS AND METHODS

### Bacterial strains, phages, and media.

Bacteria and phage were incubated at 37°C with aeration (Table 1). The WT ETEC strain EK99-F41 was used as a host to proliferate JS09 as previously described (33). Phage JS09 belongs to the family *Myoviridae* with a genome size of 169.148 kb (GenBank accession number KF582788) and was isolated by our lab (33). Luria-Bertani (LB) medium was used for bacterial liquid cultures, and LB with additional 0.6% or 1% (wt/vol) agar was used to make soft-agar plates. When necessary, kanamycin (100 μg/ml) was added to the medium.

### Phage propagation assays.

Phage was propagated by the phage lysate method (53). Phage lysate was prepared by adding approximately 1 ml phage (10^9^ PFU/ml) to 100 μl of overnight bacterial cells culture (10^8^ CFU per ml) in 25 ml LB medium at 37°C with shaking for 6 to 8 h, and the culture was completely cleared. Then the phage lysate was collected by centrifugation (10,000 × *g*, 20 min, 4°C) and filtered through a 0.22-μm Millipore filter. The phage lysate was stored at 4°C until used. The number of phages was determined by the double agar overlay plaque assay. Phage titer was carried out by adding 100 μl of serial 10-fold-diluted phage lysate and 100 μl of bacterial cultures (10^8^ CFU/ml) into 3.6 ml warm LB containing 0.6% (wt/vol) agar. The mixture was spread over an LB agar plate, incubated overnight at 37°C, and PFU per milliliter was determined.

### Isolation of phage-resistant mutants.

The phage aerosol spray experiment was conducted in a vertical flow clean bench (length by width by height of 840 cm by 700 cm by 540 cm). First, a total of 5 groups of LB 1% (wt/vol) agar plates were placed separately on the clean bench. Every group had 3 plates. Then, 5 ml of fresh EK99-F41 cultures (10^4^ CFU/ml) was sprayed all over the clean bench. Five minutes later, 5 ml of phage JS09 lysate (10^9^ PFU/ml) was sprayed onto the bacterium-polluted bench. After 24 h, the residual viable bacterial colonies on the plates were isolated to determine the changes in phage susceptibility.

### Whole-genome sequencing of EK99-F41 and WRP.

Genomic DNA from parental bacteria EK99-F41 and mutant bacteria WRP were purified via a TIANamp bacteria DNA kit (Tiangen Biotech, Beijing, China). The genomes of EK99-F41 and WRP were sequenced by using an Illumina HiSeq 2500 platform (Illumina, San Diego, CA, USA). The sequencing was paired end of 500 bp × 2. The sequencing depth was more than 100×, and the sequencing average coverage was 99.99%. Sequences were assembled into contigs and scaffolds using SOAPdenovo software (version 2.04) (54). The nucleotide mutation sites of WRP were analyzed by mapping the sequence reads archived by genome sequencing onto the genome of the parental bacteria using MUMmer (version 3.23) and BLAT software (version 35) (55, 56). Single nucleotide polymorphisms (SNPs) were detected by SAMtools (57, 58). Mutation regions were confirmed by PCR amplification and Sanger sequencing.

### Construction and verification of *rapZ*_E227Stop_ mutant.

A markerless *rapZ*_E227Stop_ mutant was constructed from the WT EK99-F41 using λ Red homologous recombination and CRISPR-Cas9 technology by GenScript Biotech (Nanjing, China) according to the method of Jiang et al. (59). Genomic DNA from the *rapZ*_E227Stop_ mutant was purified via a TIANamp bacteria DNA kit (Tiangen Biotech, Beijing, China). The *rapZ* gene was amplified by a pair of primers, RapZ-F (5′-TTCTTAACAGGGAATGTACG-3′) and RapZ-R (5′-TTCAGATAAGCGAATCATGCCATCTCC-3′). Then, the desired mutation of *rapZ* was verified by Sanger sequencing.

For complementation, the *rapZ* promoter region was searched in the RegulonDB database (http://regulondb.ccg.unam.mx/), and the *rapZ* gene was found to be under the control of an *rpoN* operon promoter. The 18-bp upstream sequence of the *rapZ* gene (from positions +18 to 0 relative to the transcriptional start site) was predicted as a ribosome-binding site (RBP) region. Next, the *rpoN* operon promoter, RBP region, and gene coding sequences of RapZ were chemically synthesized by Genecreate Biotech (Wuhan, China), cloned into a pACYC177 vector (60), and verified by DNA sequencing. Plasmid constructions were performed in E. coli DH5α. The recombinant plasmid was subsequently transformed into the *rapZ*_E227Stop_ mutant, yielding the complemented mutant strain C-*rapZ*_E227Stop_. The complemented strains were confirmed using PCR and sequence analysis. The characteristics of the strains and the plasmids are presented in Table 1.

### Phage plaque formation assays.

The lysis activity of phage was examined using spot tests on E. coli strains. A series of exponential-phase cultures were prepared for each strain. Two hundred microliters of cultures were added into 10 ml molten (45°C ≤ temperature ≤ 50°C) LB medium with agar (1% [wt/vol]). The mixture was then vortexed and poured into individual plates to produce LB agar plates. When the medium solidified, the serial dilutions of phage stock (5 × 10 ^10^ PFU/ml, 3 μl) were spotted onto the surface of the plates. The plates were inverted, incubated at 37°C overnight, and then examined and photographed.

### Phage adsorption assays.

For the adsorption efficiency of phage, E. coli strains were cultured overnight. Approximately 6.4 × 10^5^ PFU/ml of JS09 in 100 μl was mixed with a 1-ml sample of exponential-phase cultures of bacteria (10^8^ CFU/ml). The suspension was incubated at 37°C for 15 min and centrifuged at 10,000 × *g* for 1 min, after which, the phage titers remaining in the supernatant (i.e., unadsorbed phage) were determined by the double agar overlay plaque assay with EK99-F41. Adsorption efficiency was calculated by dividing the number of adsorbed phages by the initial number of phages. Each assay was performed in duplicates and repeated three times.

The adsorption kinetics assay of phage to the bacterial strains was performed according to the method mentioned above. The samples were allowed to incubate for 2, 5, 10, and 15 min at 37°C and then were centrifuged at 10,000 × *g* for 1 min to remove the cells in the mixture. The phage titers of the supernatant were immediately determined by the double agar overlay plaque assay. The experiment was carried out in triplicates.

### Phage DNA labeling and microscope absorption assay.

Phage DNA labeling and the microscope absorption assay were conducted according to the methods described previously with some modifications (11, 61, 62). Three hundred microliters of phage lysate (approximately 10^10^ PFU/ml) was mixed with 2 μg/ml 4, 6-diamidino-2-phenylindole (DAPI) (Solarbio) for 10 min. Labeled phages were then filtered through a 10-kDa ultrafiltration spin device (Millipore) at 1,500 × *g* for 90 min to remove the free DAPI. For the phage microscopic adsorption assay, 500 μl of mid-log-phase growing cells were mixed with 15 μl labeled phages, incubated at room temperature for 20 min, and centrifuged for 4 min (6,644 × *g*, 25°C) to pellet the cells. The supernatant was removed, and then cells were washed once with 1× phosphate-buffered saline (PBS), suspended in 100 μl 1× PBS, and observed and imaged at ×100 magnification under a Zeiss Scope A1 epifluorescence microscope (Axio Scope A1; Carl Zeiss, Germany). The assay was performed in triplicates.

### Bacterial growth kinetics assays.

Bacterial growth was monitored by measuring the OD_600_. Five milliliters of bacterial cultures (10^7^ CFU/ml, OD_600_ of ∼0.6) was incubated in a 10-ml glass test tube. All test tubes were incubated with shaking at 180 rpm at 37°C. The OD_600_ of each culture was monitored every hour for 10 h of incubation by using an Ultrospec 10 cell density meter (GE Healthcare, San Diego, CA, USA). The experiment was carried out in triplicates.

### Phage infectivity assays.

The infectivity of phage JS09 against bacteria in liquid was performed at multiplicities of infection (MOIs) of 100 and 10. Bacterial cultures (10^7^ CFU/ml, OD_600_ of ∼0.6) were inoculated with fresh phage stock (5 × 10^10^ PFU/ml) to achieve MOIs of 100 and 10 in 10-ml glass test tubes. The total volume of each mixture was 5 ml. All test tubes were incubated with shaking at 180 rpm at 37°C. The OD_600_ of all cultures was monitored every hour for 10 h using an Ultrospec 10 cell density meter (GE Healthcare, San Diego, CA, USA). The assays were carried out in triplicates.

For the efficiency of plating (EOP) of phage on different strains, WT EK99-F41, the designated mutant strains, and the C-*rapZ*_E227Stop_ strain were infected with phage JS09 at the same PFU, and phage titers were monitored by the double agar overlay plaque assay 6 h postinfection. EOP was calculated by dividing the phage titer obtained on each mutant strain and the C-*rapZ*_E227Stop_ strain by the phage titer on the WT strain. The experiment was carried out in triplicates.

### Scanning electron microscopy assays.

The morphological images of the bacterial cells were observed and recorded by scanning electron microscopy (Carl Zeiss/EVO LS10 scanning electron microscope; Zeiss Microscopy Ltd., Cambridge, UK). One milliliter of E. coli cells (10^8^ CFU/ml) was washed with 1 ml of PBS (pH 7.4) three times. The bacterial suspensions were centrifuged, resuspended, and fixed with 2.5% glutaraldehyde overnight at 4°C. The fixed cells were collected and washed once with 0.9% NaCl. Then, the cells were treated sequentially with increasing ethanol solutions, including 30, 50, 70, 80, 90, and 100%, for 15 min for each dehydration step. The samples were dropped onto 0.25- by 0.25-cm coverslips for drying at room temperature for 4 h and coated with gold. SEM images were taken with the microscope in high-vacuum mode at an acceleration voltage of 10 kV. The length and width of the cells were analyzed with ImageJ (https://imagej.nih.gov/ij/download.html). Forty cells of each indicated strain were measured for the analysis.

### Assays of LPS profile.

LPS was extracted from 2 ml of each bacterial culture at the same concentration of 10^9^ CFU/ml using a lipopolysaccharide extraction kit (iNtRON Biotechnology Ltd., South Korea) according to the manufacturer’s protocol. The extracted LPS was dissolved in 40 μl 10 mM Tris-HCl buffer (pH 8.0) for further assays. For LPS profile assays, 4 to 12% SDS-polyacrylamide gel electrophoresis (PAGE) of LPS was performed and visualized by silver staining as previously described (35).

### Effect of polymyxin B on adsorption of phage to LPS.

To determine if LPS is the receptor of phage JS09, the adsorption efficiency method was used, adapted from a previous report with modifications (36–38). One hundred microliters of double-distilled water containing 250 μg/ml PMB was added to 900 μl of WT EK99-F41 cell suspension containing approximately 5 × 10^8^ CFU/ml, and the mixture was incubated at 37°C for 1 h. One sample received 100 μl of double-distilled water as the control. Then, 100 μl of phage JS09 (approximately 7.5 × 10^3^ PFU/ml) was added. Adsorption efficiency was measured by the above-mentioned phage adsorption assay method.

Twenty microliters of the extracted LPS was treated with 2.3 μl of double-distilled water containing 250 μg/ml PMB (final concentration, 25 μg/ml), and the mixture was incubated at 37°C for 1 h. One LPS sample received 2.3 μl of double-distilled water as the control. Then, the mixture was added to 100 μl of JS09 suspension (approximately 6.6 × 10^3^ PFU/ml). After 15 min of incubation at 37°C, the adsorption efficiency was measured by the above-mentioned phage adsorption assay method.

### Effect of LPS on phage infection.

Effects of LPS on phage infection were evaluated by efficiency of plating (EOP) according to the method of Xiong et al. (18). Ten microliters of the extracted LPS (as described above) was added to 100 μl of phage suspension (9 × 10^3^ PFU/ml) and incubated at 37°C for 2 h. The mixture was added to 100 μl of an exponentially growing culture of WT EK99-F41 (10^9^ CFU/ml) and then mixed with 3.6 ml of 0.6% (wt/vol) soft agar and poured onto LB plates. After incubation overnight, phage titers were monitored by the double agar overlay plaque assay. Phage JS09 with LB was used as the blank control (phage titer, 100%). The experiment was carried out in triplicates and repeated three times.

### Quantitative Real-Time RT-PCR.

The expression levels of *glmS* gene were investigated using quantitative real-time PCR (qRT-PCR). Briefly, total RNA was extracted using a spin column bacterial total RNA purification kit (Sangon Biotech, Shanghai, China), followed by cDNA synthesis using the HiScript III reverse transcription (RT) supermix for qPCR kit (with a genomic DNA [gDNA] wiper) (Vazyme, Nanjing, China) according to the manufacturer’s protocol. qRT-PCR was conducted using a SYBR Premix Ex *Taq* II kit (TaKaRa, Dalian, China) and gene-specific primers (Table 3). Data analysis was conducted according to the comparative threshold cycle (2^−ΔΔ^*^CT^*) method (63) via normalization to the expression of the reference gene *rpoD*. The relative expression level is shown as the ratio to that of WT EK99-F41. The experiment was carried out in triplicates and repeated three times.

**TABLE 3:** Primers for qRT-PCR

Primer	Sequence (5′→3′)	Target gene	Product size (bp)
rpoD RT-F	AATGCTCCGTTGCTGAATATCCG	*rpoD*	221
rpoD RT-R	GTCGTCATCGCCATCTTCTTCG	*rpoD*	221
glmS RT-F	GTGACCCGTCGCTTTATCTTCC	*glmS*	233
glmS RT-R	ACCTGACCGTGGCTGATGC	*glmS*	233

### Phylogenetic tree construction.

Protein sequences of RapZ for various bacteria were obtained through UniProtKB. The sequences were aligned using ClustalW, and the phylogenetic tree was constructed using Molecular Evolutionary Genetics Analysis (MEGA) version 6.0 (64). Using the neighbor-joining method, a bootstrap consensus phylogenetic tree from 1,000 bootstrap replications for tree construction was selected. The selected numbers of bootstraps are shown on the selected branches.
